# LIS1 and NDEL1 Regulate Axonal Trafficking of Mitochondria in Mature Neurons

**DOI:** 10.3389/fnmol.2022.841047

**Published:** 2022-04-07

**Authors:** Jai P. Pandey, Liang Shi, Remi A. Brebion, Deanna S. Smith

**Affiliations:** Department of Biological Sciences, University of South Carolina, Columbia, SC, United States

**Keywords:** axon, mitochondria, dynein, DRG, sciatic nerve, LIS1, NDEL1

## Abstract

Defective mitochondrial dynamics in axons have been linked to both developmental and late-onset neurological disorders. Axonal trafficking is in large part governed by the microtubule motors kinesin-1 and cytoplasmic dynein 1 (dynein). Dynein is the primary retrograde transport motor in axons, and mutations in dynein and many of its regulators also cause neurological diseases. Depletion of LIS1, famous for linking dynein deregulation to lissencephaly (smooth brain), in adult mice leads to severe neurological phenotypes, demonstrating post-developmental roles. LIS1 stimulates retrograde transport of acidic organelles in cultured adult rat dorsal root ganglion (DRG) axons but findings on its role in mitochondrial trafficking have been inconsistent and have not been reported for adult axons. Here we report that there is an increased number of mitochondria in cross-sections of sciatic nerve axons from adult LIS1^+/–^ mice. This is probably related to reduced dynein activity as axons from adult rat nerves exposed to the dynein inhibitor, ciliobrevin D also had increased numbers of mitochondria. Moreover, LIS1 overexpression (OE) in cultured adult rat DRG axons stimulated retrograde mitochondrial transport while LIS1 knockdown (KD) or expression of a LIS1 dynein-binding mutant (LIS1-K147A) inhibited retrograde transport, as did KD of dynein heavy chain (DHC). These findings are consistent with our report on acidic organelles. However, KD of NDEL1, a LIS1 and dynein binding protein, or expression of a LIS1 NDEL1-binding mutant (LIS1-R212A) also dramatically impacted retrograde mitochondrial transport, which was not the case for acidic organelles. Manipulations that disrupted retrograde mitochondrial transport also increased the average length of axonal mitochondria, suggesting a role for dynein in fusion or fission events. Our data point to cargo specificity in NDEL1 function and raise the possibility that defects in the LIS1/NDEL1 dynein regulatory pathway could contribute to mitochondrial diseases with axonal pathologies.

## Introduction

Because of its role in human brain development LIS1 has been studied by developmental neuroscientists for over two decades (Reiner et al., 1993; Hirotsune et al., 1998; Smith et al., 2000; Saillour et al., 2009; Reiner and Sapir, 2013; Markus et al., 2020). Dominant mutations in the gene encoding LIS1, *PAFAH1B1*, cause lissencephaly (smooth brain; Wynshaw-Boris, 2007; Saillour et al., 2009). Although rare, lissencephaly is a devastating disorder characterized by severe cognitive and motor impairment, worsening seizures, and childhood lethality. Preclinical studies have demonstrated that decreasing LIS1 protein expression disrupts the timing of mitosis and neural differentiation as well as the migration of neural precursors in the developing brain, and LIS1^+/–^ mice have been used as a model to study cellular functions of LIS during brain development (Hirotsune et al., 1998; Wynshaw-Boris, 2007; Hebbar et al., 2008a; Yingling et al., 2008; Hippenmeyer et al., 2010; Moon et al., 2014; Xiang and Qiu, 2020). Several studies indicate that the expression of LIS1 in these mice is reduced by around 50% (Yan et al., 2003; Hebbar et al., 2008a; Sebe et al., 2013; Toba et al., 2013). Excessive LIS1 expression is also deleterious in both humans and mice, so LIS1 expression levels must be precisely regulated (Bi et al., 2009).

LIS1 is highly conserved across animal and fungal species, and all tested orthologs, from yeast to human, bind directly to dynein’s two heavy chains (DHCs); DHCs hydrolyze ATP and contact microtubules for processive cargo transport (Xiang and Qiu, 2020). Our cell-based studies led us to hypothesize that mammalian LIS1 stimulates dynein activity, and in our hands, purified LIS1 modestly but consistently increased the ATPase activity of purified bovine brain dynein (Smith et al., 2000; Mesngon et al., 2006; Hebbar et al., 2008b; Pandey and Smith, 2011; Hines et al., 2018). However, other studies contradicted this, and substantial data, especially using *in vitro* assays, indicated that LIS1 inhibits dynein; see Markus et al. (2020). The story remained confusing until recently when new studies refined the consensus view of how LIS1 regulates the motor. Large, multi-subunit dynein complexes can adopt a closed, autoinhibited conformation (the phi particle) or an open conformation (Zhang et al., 2017). Other dynein subunits interact with dynactin and one of several cargo adaptors, both of which are required for robust processive movement of mammalian dynein along microtubules—dynactin and activating cargo adaptors only interact with the open form, and the tripartite Dynein/Dynactin/Adaptor complex is referred to as the DDA complex (Reck-Peterson et al., 2018; Urnavicius et al., 2018; Olenick and Holzbaur, 2019; Canty and Yildiz, 2020; Lee et al., 2020; Xiang and Qiu, 2020). The newest model is that LIS1 binding to DHC stabilizes the open conformation, resulting in a higher proportion of motors able to form active DDA complexes (Qiu et al., 2019; Elshenawy et al., 2020; Htet et al., 2020; Marzo et al., 2020; McKenney, 2020). Moreover, LIS1 promotes the formation of adaptor complexes containing two motors (D_2_DA complexes) which generate more force and likely have longer processive runs (Elshenawy et al., 2020; Htet et al., 2020).

Dynein is the primary motor driving retrograde transport in axons (Guedes-Dias and Holzbaur, 2019). Our studies using LysoTracker to label acidic organelles in cultured adult rat and mouse sensory neurons showed that increasing the ratio of LIS1 to dynein stimulated retrograde axonal transport, while a missense LIS1 mutation that prevents LIS1 from binding to dynein (LIS1-K147A) severely disrupted retrograde transport, as did an shRNA targeting LIS1 (Pandey and Smith, 2011). LIS1 and dynein interact with NDEL1, and a homolog, NDE1 (Niethammer et al., 2000; Sasaki et al., 2000; Bradshaw and Hayashi, 2017). In some cases, phosphorylated NDEL1 appears to work with LIS1 to stimulate dynein (Niethammer et al., 2000; Sasaki et al., 2000; Hebbar et al., 2008b; Pandey and Smith, 2011; Klinman et al., 2017). However, in cultured DRG axons, NDEL1 KD or expression of a LIS1 missense mutation that blocks NDEL1 binding but not dynein binding (LIS1-R212A) had only a minimal impact on retrograde transport of acidic organelles (Pandey and Smith, 2011). Moreover, no increase in ATPase activity was observed when NDEL1 was included in our ATPase assay (unpublished results). Others reported that LIS1 reduced ATPase activity (Yamada et al., 2008). To our knowledge, the role of NDEL1/NDE1 in the DDA/D_2_DA complex is not yet known.

Inducing LIS1 depletion in 2-month-old mice resulted in the rapid onset of neurological phenotypes and death, demonstrating a critical postdevelopmental role (Hines et al., 2018). The most likely cause was disrupted sensorimotor and cardiorespiratory circuits, possibly due to altered axonal transport. The adult nervous system is uniquely dependent on a continuous energy supply for synaptic function, and mitochondrial trafficking abnormalities are clearly associated with later onset neurological disease (Misgeld and Schwarz, 2017; Castora, 2019; Mandal and Drerup, 2019; Rangaraju et al., 2019; Han et al., 2020; Yang et al., 2021). Because of this, we reasoned that a disruption in mitochondrial dynamics could be involved in the phenotype. We have now carried out MitoTracker transport studies in adult DRG cultures and found clear roles for both LIS1 and NDEL1. We uncovered a surprising difference between acidic organelles and mitochondria with respect to NDEL1. We also found LIS1 and NDEL1 perturbations that reduce trafficking increase mitochondrial size, suggesting that these regulators play a role in fission or fusion events.

## Methods

### Electron Microscopy of Sciatic Nerve Sections

#### LIS1^+/–^ Nerves

Sciatic nerve segments were dissected from adult LIS1^+/–^ mice and fixed overnight in 2.5% glutaraldehyde at 4°C. Fixed nerves were sent to the electron microscopy core facility at the UofSC school of medicine for processing and electron microscopy using a JEOL 1400 plus system, (Peabody, MA, USA).

#### Ciliobrevin-D Releasing Nerve Cuffs

PEVA (polyethylene vinyl acetate, Sigma Aldrich, St. Louis, MO, USA, #340502) nerve cuffs were used to release a small molecule inhibitor of dynein, ciliobrevin-D (EMD Millipore, Burlington, MA, USA # 25041) directly onto the sciatic nerve of adult rats (Smith and Skene, 1997; Schneider et al., 2017). Ciliobrevin-D was dissolved in DMSO and added to a 10% solution of PEVA dissolved in methylene chloride to a final concentration of 1 mM. This requires heating to 60°C. DMSO only was used to make control cuffs. The liquid PEVA/drug mixture was poured into a flat mold, flash-frozen on dry ice, and the solvent evaporated with anhydrous calcium sulfate at −20°C for 1 week. The solid yet pliable sheets were cut into strips, 1 mm wide, and wrapped around the exposed sciatic nerve at mid-thigh. Ends were superglued together, then the muscle and skin are sutured, and the cuffs left in place for 48 h, after which nerve segments under the cuffs were dissected and fixed overnight in 2.4% glutaraldehyde at 4°C. Sections were analyzed blinded, in that the person counting mitochondria in axonal cross-sections did not know whether sections were from LIS1^+/–^ mice or wild-type (WT) littermates or from the animals exposed to ciliobrevin-D or DMSO only.

### Immunoprecipitation and Western Blotting of Nerve Extracts

Sciatic nerve segments were removed 48 h after cuff application. Nerves were Dounce-homogenized in ice cold 50 mM Tris, pH 8.0, 250 mM NaCl, 0.5% NP40, and halt protease and phosphatase inhibitor cocktail (ThermoFisher, Waltham, MA, USA, 78440). Homogenates were centrifuged at 30,000× *g* for 30 min. Supernatants were split into two new tubes and immunoprecipitated with anti-dynein intermediate chain antibody coupled to agarose (Santa Cruz Biotechnology Inc., USA, sc-13524 AC) or normal mouse IgG coupled to agarose (Santa Cruz Biotechnology Inc., USA, sc-2343) for 2 h at 4°C with rocking. Beads were washed three times in lysis buffer and precipitated proteins separated by 10% acrylamide SDS-PAGE and transferred to PVDF membrane for probing with the dynein intermediate chain antibody (Santa Cruz Biotechnology Inc., USA, sc-13524).

### RNAi and Mammalian Expression Constructs and Antibodies

Complementary hairpin sequences to *LIS1*, *DYNC1H1 (DHC)* and* NDEL1* in the pSil-EGFP vector (RRID:Addgene_52675) were provided by LH Tsai (MIT) and have been well characterized (Shu et al., 2004; Hebbar et al., 2008b; Pandey and Smith, 2011; Klinman and Holzbaur, 2015). *LIS1*: GAGTTGTGCTGATGACAAG (1,062–1,080 bp), *DYNC1H1*: GAAGGTCATGAGCCAAGAA (9,753–9,771 bp), *NDEL1*: GCAGGTCTCAGTGTTAGAA (276–294 bp). Scrambled sequences were generated from these sequences and have been shown to have no effect on the expression of the relevant proteins. These scrambled sequences were used as controls for each specific shRNA. To generate HA-LIS1, HA-LIS1-K147A, and HA-LIS1-R212A, full-length murine LIS1 and point mutants of LIS1 (provided by A. Musacchio) were subcloned into a pCruzHA vector (Santa Cruz Biotechnology, Inc., Dallas, TX, USA). Axons were labeled for dynein using the dynein intermediate chain 74.1 mouse monoclonal antibody (Santa Cruz Biotechnology Inc., Dallas, TX, USA, sc-13524).

### DRG Culture and Transfection

Primary cultures of sensory neurons from 4 to 6 lumbar DRG of adult rats were prepared as described in (Smith and Skene, 1997), with a few minor changes. Briefly, neurons in 3-month-old male Sprague Dawley rats were conditioned for rapid axon elongation by sciatic nerve crush 48 h prior to harvesting. This allows long axons to be imaged before non-neuronal cells proliferate because mitochondria from these cells can obscure imaging of axonal mitochondria. This also allows us to avoid mitotic toxins such as cytosine arabinoside which reportedly can impact mitochondrial DNA synthesis and compromise mitochondrial function (Zhuo et al., 2018). Following enzymatic and mechanical dissociation, suspended cells were subjected to centrifugation through a 15% sucrose solution to reduce the number of smaller glial cells. Neurons were transfected immediately after dissection using the small cell number SCN Basic Nucleofector kit for primary neurons (Amaxa Biosystems, Walkersville, MD, USA, VSPI #1003). All LIS1, DHC, or NDEL1 expressing constructs were co-transfected with an EGFP vector (pEGFP-C1, Clontech, Mountain View, CA, USA, # V012024) to identify transfected neurons. Following transfection, neurons were resuspended in Hamm’s F14 medium (Biowest, Nuaille, France) supplemented with 10% horse serum (Biowest, Nuaille, France) and buffered with 25 mM HEPES, then plated onto German glass coverslips (Fisher, Waltham, MA, USA) coated with 10 μg/ml poly-D-lysine (Sigma, St. Louis, MO, USA) and 10 μg/ml laminin (EMD, Millipore, Burlington, MA, USA). Neurons were used within 3–4 days of culture for transport studies.

### Fluorescence Time-Lapse Microscopy

After exposure to 100 nM Mitotracker Red CMXRos (Invitrogen, Waltham, MA, USA, M7512) for 20 min, coverslips were transferred into a fresh medium containing 25 mM Hepes, pH 7.4, and 10 mM OxyFluor (Oxyrase, Inc OF-0005), in a water-heated custom-built microscope stage warmed to 37°C. EGFP-positive, 100 μm long axon segments that were clearly linked to a specific neuronal cell body and did not cross over other axons were selected for analysis. Axon segments were chosen that were at least 20 μm from cell bodies or growth cones because the direction of movement seemed to be different close to these regions (more anterograde near growth cones, more retrograde near cell bodies). Time-lapse microscopy was performed using an Axiovert 200 inverted microscope (Carl Zeiss Inc., Jena, Germany) equipped with C-Apo 63X/1.2 W/0.2 water-immersion objective. Digital images were acquired every 2.6 s for 4 min (92 frames) using a charge-coupled camera (AxioCam HRm, Carl Zeiss Inc., Jena, Germany) linked to AxioVision software (version 4.7, Carl Zeiss Inc., Jena, Germany).

### Analysis of Percentage of Mitochondria Moving in Axons

To determine the percentage of mitochondria moving and their direction, kymographs were generated from time-lapse movies using Image J open-source image processing software and the KymoToolBox Plugin. Cultures from three different rats were used to control for individual differences. For each condition, 12 100 μm axon segments were imaged (four per coverslip) Imaged axon segments were at least 20 μm from cell bodies, growth cones or branch points. Analyses were carried out blinded, so that the person analyzing the kymographs did not know which treatment was being analyzed. A segmented line tool was used to isolate individual organelle tracks in the kymographs. Lines that showed less than 5 μm displacement in either direction during the recording interval were categorized as static mitochondria. Lines that sloped toward the right only, with no switching at any point, with a net displacement of >5 μm were categorized as retrogradely moving mitochondria. Lines that sloped to the left only with no switching at any point, with a net displacement of >5 μm were categorized as anterograde. Mitochondria that showed net displacement of >5 μm in both directions at any point during the interval were categorized as both, and organelles that had no displacements >5 μm were categorized as static. Mitochondria typically exhibited runs of variable lengths in either direction with intermittent pauses. We deemed these runs “motile events” and focused on the analysis of retrograde motile events (RMEs). A single organelle could produce several RMEs between pauses or anterograde runs. Many of these are shorter than 5 μm, but overall retrograde displacement for an individual organelle would be the sum of the retrograde motile events, and this needed to be greater than 5 μm or it was categorized as static. These static organelles sometimes “wobble” a few microns but do not undergo sustained processive movement in either direction.

### Analysis of Average Speeds and Run Lengths of Retrograde Motile Events

ImageJ software with a manual tracking plug-in that allows retrieval of object coordinates for image frames of time-lapse series of images was used to determine run lengths and speeds. If an organelle did not reach an average speed of 0.1 μm/s during three consecutive time-lapse intervals it was taken as a pause. If the organelle moved again later, it was considered a new motile event. Average run length and velocities were determined by finding the average value for new track positions for each RME. Mean velocities of all frames in an RME were determined for each RME. The percentage of motile events with average velocities >0.5 μm/s was also determined. Retrograde flux in μm/min was calculated as the sum of net displacements of all RMEs for each of the three experiments (animals, 12 axons from each animal) divided by the recording interval time (4 min).

### Analysis of Mitochondrial Length

Mitochondria labeled with MitoTracker Red could be visualized in neurons fixed in 4% paraformaldehyde for 10 min at 37°C. Axons in three separate cultures from three different animals were analyzed. To avoid errors that might arise by measuring different axonal regions, we limited the analysis of mitochondria to long unbranched axons or to long axonal segments between branch points at least 30 μm from the branch points. We did not include mitochondria in growth cones or in axonal segments within 20 μm from the beginning of the growth cone or 30 μm from the axon hillock. Five axons segments were analyzed in each culture, and the total of 15 segments analyzed contained between 116 and 134 mitochondria.

### Statistics

GraphPad Prism 9, USA was used to determine statistical significance for percent retrograde, run lengths, and speeds, using nonparametric one-way ANOVA with a Kruskal Wallis test. For all quantified results, except retrograde flux, Dunn’s multiple comparisons test was used and the false discovery rate was controlled for using the two-stage step-up method of Benjamini, Krieger, and Yekutieli. Statistical significance for retrograde flux was determined by one-way ANOVA with Tukey’s multiple comparisons test. The bootstrap sampling distributions shown below the scatter plots were generated using estimationstatistics.com (Ho et al., 2019) for a shared control (either scrRNA, EGFP, or both) Each mean difference is depicted as a dot. Each 95% confidence interval is indicated by the ends of the vertical error bars.

## Results

### Altered Mitochondrial Distribution in the Sciatic Nerve Caused by Reduced LIS1 Expression or Pharmacological Inhibition of Dynein

LIS1^+/–^ mice have often been used to study the cellular consequences of LIS1 haploinsufficiency. Although the mice experience some developmental delay and have hyperexcitability in some brain regions, adult mice appear healthy, reproduce normally, and do not show signs of neurodegeneration such as leg clasping upon tail suspension. We did not detect fewer axons in adult LIS1^+/–^ mouse sciatic nerves, and adult DRG neurons from LIS1^+/–^ animals appeared to extend normal axons in culture compared to those from LIS1^+/+^ littermates (Pandey and Smith, 2011). Total iKO in adult DRG neurons resulted in shorter axons but these were sufficiently long to carry our axonal transport studies (Hines et al., 2018).

**Figure 1:**
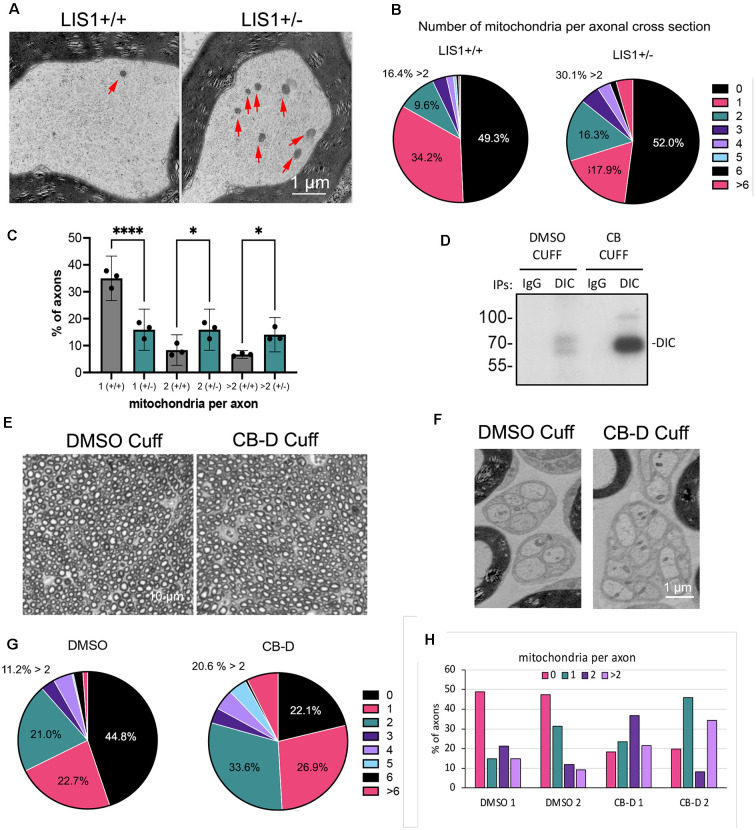
More mitochondria are observed in sciatic nerve axonal cross-section from LIS1^+/–^ mice or rat nerves exposed to Ciliobrevin-D. **(A)** Representative electron micrographs of cross-sections of myelinated axons in sciatic nerves from adult WT LIS1 and LIS1^+/–^ mice at mid-thigh. Red arrows point to mitochondria within the axoplasm. EM images from three different mice of each genotype were analyzed for numbers of mitochondria per axon. **(B)** The pie graphs show the percentage of all axonal cross-sections that contained the indicated number of mitochondria (1- >6). A total of 676 LIS1^+/+^ axons and 405^+/–^ axons from three mice of each genotype were included, approximately equal numbers per animal. The variation is not because of degeneration but simply a matter of number of axons in each EM micrograph, which was variable. **(C)** The bar graph shows the mean of data (+/– 95% CI) from the three mice in each category. Axons with 0 mitochondria were excluded because the numbers were large and the differences in one mitochondrion, two mitochondria, or >2 mitochondria were less clear. Significance determined by ANOVA using Holm-Šídák’s multiple comparisons test. ****p < 0.0001; *p > 0.05. **(D)** Ciliobrevin-D (CB-D) was used to inhibit dynein by placing PEVA nerve cuffs impregnated with the compound around adult rat sciatic nerve in mid-thigh. After 48 h dynein had accumulated under a CB-D cuff but not a DMSO control cuff as seen in the IP-Western for dynein intermediate chain (DIC). **(E)** Brightfield image of nerve sections stained for EM shows that the nerves look normal and have similar numbers of axons. **(F)** Unmyelinated axons in Remak bundles also appeared healthy in both DMSO and CB-D treated nerves. **(G)** The pie graphs show the percentage of all axonal cross-sections that contained the indicated number of mitochondria (1- >6). 150 DMSO axons and 105 CB-D axons from two rats were included. **(H)** The bar graph shows the percentage with 0, 1, 2 or >2 mitochondria for each DMSO treated nerve and each CB-D treated nerve.

To determine if mitochondria distribution is altered in adult LIS1^+/–^ axons *in situ*, electron micrographs (EMs) of sciatic nerve cross-sections from three mice (and three WT littermate controls) were examined. Figure 1A shows two myelinated axons, but we examined both myelinated and unmyelinated axons to ensure we counted both motor and sensory neurons. Around half of the axonal cross-sections in both LIS1^+/–^ and WT nerves had no visible mitochondria (Figure 1B). The percentage of axons with greater than two mitochondria per axon was increased from 16.4% in the WT nerves to 30.1% in the LIS1^+/–^ nerves. At the same time, the percentage with only one mitochondrion was reduced from 34.2% to 17.9%, and the percentage with four or more mitochondria increased from 3.4% to 8.9%. Significantly fewer LIS1^+/–^ axons had only one mitochondrion per axon (*p* < 0.0001). Significantly more had ≥2 mitochondria (*p* = 0.035; Figure 1C).

A similar trend was observed when dynein was inhibited pharmacologically, although we only tested this in two adult rats. We used pliable PEVA nerve cuffs for the release of the dynein inhibitor, ciliobrevin-D (CB-D, onto the mid-region of adult rat sciatic nerves for 48 h. Control cuffs contained only DMSO. A Western blot of dynein intermediate chain immunoprecipitated from nerve segments immediately under the cuffs demonstrated that dynein had accumulated under the CB-D cuff, but not the DMSO cuff, suggesting an interference with dynein-dependent transport in the nerve exposed to CB-D (Figure 1D). We counted the number of mitochondria per axon in EMs sections of nerve segments taken from under the cuffs. Neither the drug nor the cuffs themselves appeared to physically damage the nerve as can be seen in the low magnification images in Figure 1E. There did not appear to be significant degeneration or loss of axons. Even small unmyelinated fibers in Remak bundles appeared intact (Figure 1F). CB-D reduced the percentage of axons with no mitochondria from 42.9% to 22.1% (Figure 1G). The percentage with two or more mitochondria per axon increased from 36.2% to 58.4%. Figure 1H shows the percentages of axons with 0, 1, 2, or >2 mitochondria from all four nerves. Both CB-D-cuffed nerves showed a trend towards more mitochondria per axon (Figure 1H).

Together these data suggest that reducing LIS1 expression or inhibiting dynein pharmacologically alters mitochondrial dynamics in axons *in vivo*, but the precise mechanisms by which these manipulations cause the accumulation of mitochondria in sciatic nerve axons is not known. Inhibition of dynein by Ciliobrevin-D also impacts anterograde transport in cultured neurons, so altered anterograde or retrograde transport could contribute to the phenotype, as could changes in docking and or fission or fusion (Roossien et al., 2015; Sainath and Gallo, 2015). Dynein is important for microtubule sliding and the establishment and maintenance of uniform microtubule polarity in axons (Rao et al., 2017). Dynein inhibition could thus disrupt anterograde transport by disrupting the uniform microtubule polarity in axons. Also, if microtubules being moved anterogradely by dynein normally have attached organelles, then disrupting this process could prevent those anterograde movements.

### Both LIS1 and NDEL1 KD Reduce Motile Mitochondria Cultured Adult Rat DRG Axons

**Figure 2:**
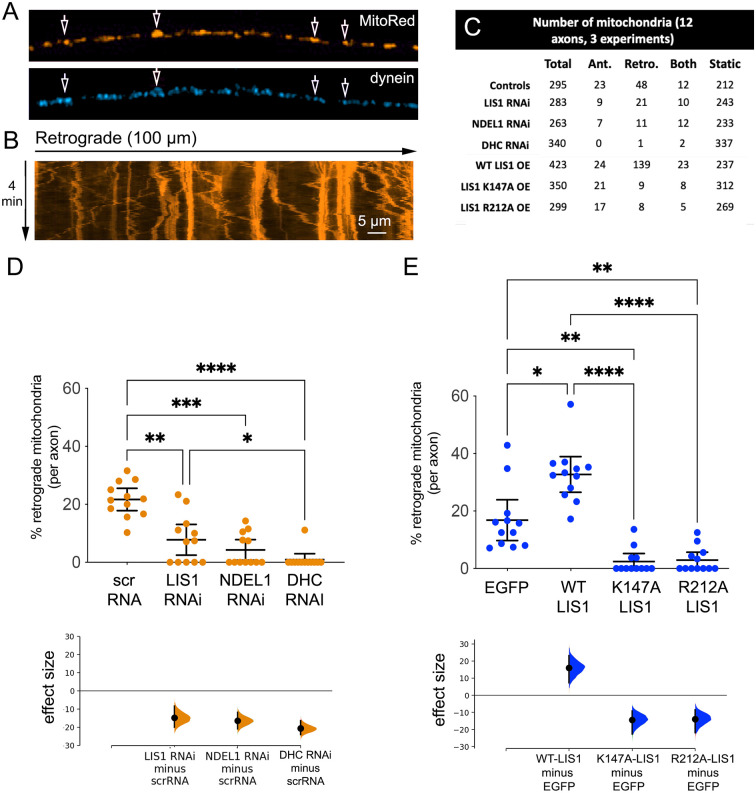
Both LIS1 and NDEL1 impact the percentage of axonal mitochondria moving retrogradely. **(A)** Axonal dynein motors stained with a dynein intermediate chain antibody (bottom panel) colocalize with axonal mitochondria (labeled with MitoTracker Red, top panel) in cultured adult rat DRG neurons. Arrows point to specific organelles to better visualize colocalization. **(B)** A typical kymograph generated from a time-lapse movie of MitoTracker Red-labeled mitochondria in a living axon. **(C)** The table shows the total numbers of mitochondria from 12 axons (in three cultures prepared from different rats) and the numbers that moved anterogradely (Ant.) retrogradely (Retro.), moved both directions (both), or remained static during the recording interval for three different KDs (LIS1, NDEL1, and DHC RNAi) and three different LIS1 constructs (WT LIS1, K147A-LIS1, 212A-LIS1). Scrambled RNA and EGFP only controls were combined since numbers were similar. **(D)** The percentage of mitochondria that only moved retrogradely in axons exposed to the indicated RNAi constructs is plotted on the upper axes. Each point corresponds to a single axon (of 12 axons measured from three independent cultures from three different rats). Error bars show the mean ± CI. The effect size is visualized on the lower axes, in which mean differences from a shared control are plotted as bootstrap sampling distributions. The mean difference is depicted as a dot and 95% confidence interval is indicated by the ends of the vertical error bars. **(E)** Similar plots are shown for axons overexpressing LIS1 constructs. For all data, significance was determined by Kruskal-Wallis nonparametric ANOVA with multiple comparisons using the two-stage linear step-up procedure of Benjamini, Kreiger, and Yekutieli to control for false discovery rate. ****p < 0.0001; ***p < 0.001; **p < 0.01; *p < 0.05.

DRG axons extend only axon-like projections, with 95% of microtubule minus-ends oriented towards the cell body (Heidemann et al., 1981; Baas et al., 1988; Kleele et al., 2014; Foster et al., 2022). Mitochondria that move retrogradely towards the cell body are very likely being conveyed by minus-end directed dynein motors. However, dynein has been observed to colocalize with mitochondria moving in both directions, and dynein and kinesin motors can bind to the same organelle (Hirokawa et al., 1990; Schwarz, 2013; Lin and Sheng, 2015). In fixed, cultured adult rat DRG axons immunofluorescently-labeled dynein can be observed near a subset of MitoTracker Red-labeled mitochondria, and so likely contributes to trafficking events in these axons (Figure 2A). After 3–4 days, kymographs generated from time-lapse movies of MitoTracker Red-labeled mitochondria in living axons (Figure 2B) were used to determine the number of mitochondria that moved retrogradely or remained static during the 4-min recording interval.

In control axons, ~10–30% underwent retrograde-only motility. To knockdown LIS1, NDEL1, or DHC, short hairpin RNAs (shRNAs) were introduced into newly dissociated neurons as described in (Pandey and Smith, 2011). Precise comparison of reduced protein levels after RNAi transfection using western blotting is difficult in adult DRG neurons because of sparse cultures and relatively low transfection efficiency. However, these same DHC, NDEL1, and LIS1 RNAi constructs reduce endogenous proteins in 3T3 cells by >60% (Shu et al., 2004), and we reported that the axonal immunofluorescence of each protein was dramatically reduced when neurons were transfected with the same shRNA constructs (Pandey and Smith, 2011). Twelve axon segments from three different DRG cultures from three different rats were analyzed for each condition. Figure 2C shows the total numbers of mitochondria in each set, and the numbers that moved anterogradely, retrogradely, switched directions one or more times, or remained static during the recording interval. KD of LIS1 and DHC reduced the numbers moving in both retrograde and anterograde directions as was the case for acidic organelles. Unexpectedly, NDEL1 KD also impacted these numbers to a much greater extent than we reported for acidic organelles. We focused on retrograde movements for the remainder of the study as these are the most likely to be driven by dynein.The percentage of retrograde mitochondria per axon after RNAi treatment is plotted in Figure 2D. LIS1 and DHC KD significantly reduced the percentage of mitochondria that underwent retrograde-only motility (*p* < 0.0001 and *p* = 0.0017, respectively). NDEL1 KD had a significant inhibitory effect (*p* = 0.0001), which was not observed for acidic organelles (Pandey and Smith, 2011). While this might be explained by differences in knockdown efficiency of the specific shRNAs in the two different studies, we used the same DRG culture protocols, the same constructs, and the same transfection method in both studies, so it impossible that NDEL1 has a different, and possibly more critical, role in mitochondrial transport than in transport of acidic organelles.

### OE of WT-LIS1, but Not Dynein- or NDEL1-Binding Mutants, Increases the Percentage of Mitochondria Moving Retrogradely in Adult Rat DRG Axons

OE of WT LIS1 increased the number of mitochondria moving retrogradely or in both directions but did not increase the number moving anterogradely (table, Figure 2C, and *p* = 0.03, Figure 2E). Expression of the dynein-binding mutant, LIS1-K147A, was not able to stimulate retrograde transport, and as with acidic organelles, significantly reduced the percentage of retrograde mitochondria during the recording interval (*p* = 0.001, Figure 2E). Again surprisingly, the NDEL1-binding mutant LIS1-R212A was very effective at reducing the percentage of retrograde mitochondria (*p* < 0.0001), in stark contrast to that observed for acidic organelles labeled with LysoTracker. Interestingly neither mutant has a strong impact on the numbers of anterograde organelles (Figure 2C). Together the over expression data supports the idea that NDEL1 is uniquely involved in the dynein-dependent transport of mitochondria in axons.

### LIS1 and NDEL1 shRNAs Shorten Run Lengths and Reduce Velocities of Retrograde Motile Events (RMEs)

**Figure 3:**
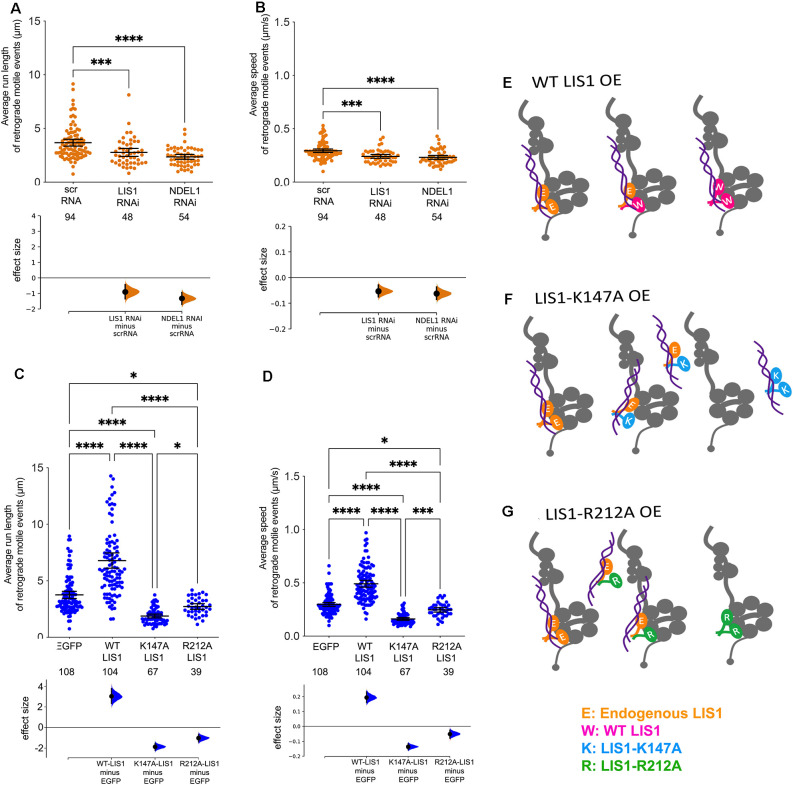
Both LIS1 and NDEL1 impact the run lengths and average speeds of retrograde motile events (RMEs). **(A–D)** Average run lengths and average velocities of RMEs of axonal mitochondria were determined using manual tracking software. The data is from 12 axon segments imaged in DRG cultures from three different adult rats (four axons from each rat). Each axon segment typically had 10–30 mitochondria, some of which moved during the recording interval. A single mitochondrion can undergo more than one RME. The numbers of individual RMEs measured in all 12 axon segments per condition are shown below the X-axis labels. Run lengths **(A)** and average speeds **(B)** are significantly reduced by RNAi-mediated KD of LIS1, NDEL1, or DHC. The raw data is plotted on the upper axes. Each point corresponds to a single axon (of 12 axons measured from three independent cultures from three different rats). Error bars show the mean ± 95% confidence interval.The effect size is visualized on the lower axes, in which mean differences from a shared control are plotted as bootstrap sampling distributions. Mean difference is depicted as a dot and 95% confidence interval is indicated by the ends of the vertical error bars. OE of WT LIS1 significantly increases both the run lengths **(C)** and average speeds **(D)** of RMEs, while both LIS1-K147A or LIS1-R212A significantly reduce run lengths and average speeds. For all data, significance was determined by Kruskal-Wallis nonparametric ANOVA with multiple comparisons using the two-stage linear step-up procedure of Benjamini, Kreiger and Yekutieli to control for false discovery rate. ****p < 0.0001; ***p < 0.001; *p < 0.05. **(E–G)** Schematics depicting potential impacts of OE of different LIS1 constructs. **(E)** WT-LIS1 (W, pink) could form homodimers and heterodimers with endogenous LIS1 (E, orange) all of which are likely able to stimulate dynein. **(F)** LIS-K147A (K, blue) homodimers should not be able to bind to and stimulate dynein but may sequester NDEL1 away from endogenous LIS1/dynein complexes. Heterodimers with endogenous LIS1 may partially bind to dynein and NDEL1, but given our results, this likely to exert dominant negative effects on dynein activation. **(G)** LIS1-R212A (R, green) homodimers may bind to dynein but should not be complexed with NDEL1. Heterodimers with endogenous LIS1 may only partially bind to dynein and NDEL1, again likely exerting dominant negative effects.

We used manual tracking software to measure distinct “motile events” that occurred between pauses or directional changes. A single organelle can undergo multiple motile events during the recording interval. These can be in either direction and/or be separated by pauses. Run lengths and average speeds are easily calculated from the manual tracking data. Both LIS1 KD and NDEL1 KD significantly reduced average run lengths (*p* = 0.0002, and *p* < 0.0001, respectively) and the average speeds (*p* = 0.0001, and *p* < 0.0001, respectively) for individual RMEs (Figures 3A,B). The manual tracking data allowed us to calculate overall retrograde flux (RF) which is the sum of retrograde displacements of all organelles (263–340) in all axons (12) divided by the 4-min recording interval time). LIS1 and NDEL1 KD significantly reduced RF. The control RF was 4.6 μm/min. RF after LIS1 KD was 1.8 μm/min (*p* = 0.0013) and after NDEL1 KD 1.7 μm/min (*p* = 0.0011).

### OE of WT-LIS1, but Not Dynein- or NDEL1-Binding Mutants, Increases Run Lengths and Velocities of Retrograde Motile Events

OE of WT LIS1 significantly increased total RME run lengths (*p* < 0.0001), while neither of the two mutant proteins were able to do so (Figure 3C). LIS1-K147A and LIS1-R212A NDEL1 both significantly reduced run lengths compared to the controls (*p* < 0.0001 and *p* = 0.036, respectively). A similar pattern was observed with average speeds (*p* < 0.0001 and *p* = 0.034, respectively, Figure 3D). 70.97% of RMEsmeasured in LIS1 OE axons reached speeds of 0.5 μm/s or greater, compared to just 26.85% in control axons. Again LIS1-K147A and LIS1-R212A reduced this (K147A, 1.47% R212A, 7.5%) WT LIS1 OE also significantly increased RF (9.4 ± 2.1 μm/min compared to 5.4 ± 0.7 μm/min for the control; *p* = 0.010). LIS1-K147A and LIS1-R212A both significantly reduced RF (K147A, 1.68 ± 0.4 μm/min, *p* = 0.0152; R212A, 1.46 ± 0.4 μm/min, *p* = 0.0110).

### LIS1 and NDEL1 Impact the Length of Individual Mitochondria in DRG Axons

**Figure 4:**
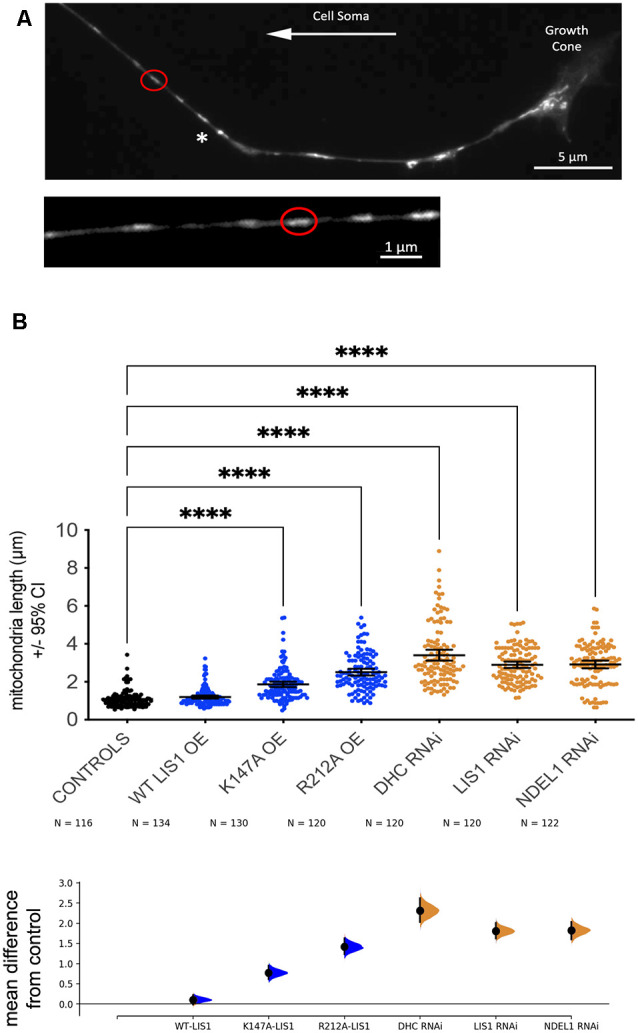
Manipulations that reduce retrograde transport of mitochondria also cause an increase in the length of axonal mitochondria. **(A)** Axonal mitochondria were visualized using mitotracker red in fixed cultures of adult rat DRG neurons. The lengths of individual mitochondria were measured in axon shafts at least 30 μm from cell bodies, branch points or growth cones. The lower panel shows a digital enlargement of the axon region the top panel that contains the circled mitochondrion. **(B)** Size measurements are shown for the indicated groups. Total numbers of mitochondria measured are shown below the X-axis labels. The raw data is plotted on the upper axes. Each point corresponds to a single axon (of 15 total from three independent cultures from three different rats). Error bars show ± 95% confidence interval. The effect size is visualized on the lower axes, in which mean differences from a shared control are plotted as bootstrap sampling distributions. Mean difference is depicted as a dot and a 95% confidence interval is indicated by the ends of the vertical error bars. ScrRNA and EGFP controls were combined because they were similar. Expression of LIS1-K147A or LIS1-R212A significantly increased mitochondrial lengths, as did RNAi targeting DHC, LIS1, or NDEL1. WT LIS1 OE did not significantly impact lengths. Significance was determined by Kruskal Wallis nonparametric ANOVA with multiple comparisons using the two-stage linear step-up procedure of Benjamini, Kreiger and Yekutieli to control for false discovery rate. ****p < 0.0001.

Most mitochondria labeled with Mitotracker Red in axons appear as discreet organelles whose length parallel to the axon is greater than their width (Figure 4A). This is particularly true in the mid axon region that is not adjacent to cell bodies, growth cones, or branch points. We measured the lengths of over 100 individual mitochondria per condition in axonal mid-regions. Surprisingly, mitochondria were significantly longer under conditions that reduced retrograde transport parameters (*p* < 0.0001 in each case, Figure 4B). These conditions included LIS- K147A and LIS1-R212A OE as well as LIS1 and NDEL1 KD. DHC KD also increased mitochondria lengths, supporting the idea that reduced dynein-dependent transport is involved in this size change. Interestingly, WT LIS1 did not significantly impact mitochondrial length, which suggests that normal axonal dynein activity is sufficient for maintaining normal lengths, while reducing axonal dynein activity is disruptive to normal regulation.

## Discussion

This work supports a model in which both LIS1 and NDEL1 positively impact dynein-dependent retrograde mitochondrial transport and potentially play a role in fission and fusion events. The finding that reduced LIS1 expression in mice or inhibition of dynein in rats resulted in more mitochondria in the sciatic nerve cross-section might reflect reduced overall retrograde and anterograde transport. Dynein colocalizes with mitochondria moving in either direction so it is likely that kinesin and dynein coordinate the transport of individual mitochondria (Hirokawa et al., 1990; Lin and Sheng, 2015). In COS-7 cells TRAK2 can stimulate kinesin-driven anterograde transport of mitochondria and dynein-dependent retrograde transport (Fenton et al., 2021). There are also several studies in which inhibiting one motor leads to reduced transport in both directions (Hancock, 2014), as we observed for dynein or LIS1 KD in cultured sensory neurons. However, this might not be the case *in vivo*. Unopposed anterograde transport of mitochondria in sciatic nerve axons could theoretically account for the increased numbers. Alternatively, more mitochondria might be apparent per cross sections if LIS1 loss or dynein inhibition causes individual mitochondria to be longer *in vivo*. Finally, reducing LIS1 expression or dynein function might result in upregulation of local anchoring of mitochondria by syntaphilin, which was recently shown to be modulated by the LIS1 and NDEL1 interacting protein disrupted in schizophrenia (DISC1; Park et al., 2016). While the trend towards increased mitochondria is similar between the LIS1^+/–^ nerves and the CB-D cuffed nerves, it is interesting that the mitochondria number in LIS1^+/+^ and DMSO control nerves are different. This could reflect differences in animal size, with rats potentially requiring more dynamic mitochondria, but we cannot rule out that the cuffs are having an effect. Future studies will be needed to determine the mechanism behind our *in vivo* observations.

Cellular toxicity or altered microtubule dynamics caused by reduced dynein activity could indirectly result in altered mitochondrial dynamics in our studies. However, axons did not appear to be degenerating and cell numbers were not reduced, so we have therefore interpreted our results in the context of recent models for LIS1 regulation of dynein. For example, LIS1 KD likely reduces the proportion of “open” dynein available to form active DDA and D_2_DA complexes with mitochondrial cargo adaptors like TRAK and MIRO (Birsa et al., 2013; Fenton et al., 2021). In this scenario, a greater number of dynein motors would be non-processive, and fewer processive motors would contain multiple dynein motors. The former could explain the reduced numbers of retrogradely moving mitochondria, and the latter could explain the shorter/slower RMEs. In contrast, OE of WT-LIS1 would increase the proportion of active DDA and D_2_DA complexes, explaining the increase in numbers of retrogradely moving mitochondria as well as longer/faster RMEs. A recent study showed that dynein-mediated transport of TRAK2, a cargo adaptor for mitochondria, is minimal unless LIS1 is present at a sufficient level, further supporting a role for LIS1 in mitochondria transport (Fenton et al., 2021). We recognize that WT-LIS1 OE might stimulate retrograde mitochondrial flux by another mechanism, such as sequestering dynein inhibitors, but to date, this has not been demonstrated. It is telling that a LIS1 isoform with a missense mutation that blocks its interaction with dynein not only was unable to stimulate retrograde mitochondrial flux but significantly reduced flux. LIS1-K147A retains its ability to homodimerize and interact with NDEL1, indicating that it is properly folded, and suggesting a possible mechanism for its dominant negative impact. Indeed, the expression of the mutant blocked the ability of dynein to coprecipitate endogenous LIS1 and reduced its ability to coimmunoprecipitate NDEL1 (Pandey and Smith, 2011).

One fascinating aspect of our study is the finding that both NDEL1 KD and the expression of LIS1-R212A, a mutant that retains its ability to bind to dynein but not to NDEL1, dramatically inhibited retrograde mitochondria transport dynamics. It is not clear how the presence of NDEL1 (or NDE1) impacts the formation of DDA and D_2_DA complexes, but our data suggest it might be relevant, at least in the case of mitochondria, as the NDEL1 manipulations used in this study had minimal impact on acidic organelles labeled with LysoTracker. Our study places LIS1 as a positive dynein regulator with respect to mitochondria in adult rat sensory neurons, which is consistent with an earlier report of LIS1 KD in embryonic hippocampal neurons (Shao et al., 2013). In that study LIS1 KD was much more impactful on mitochondrial transport than NDEL1 KD, so developmental age or regenerative state could impact mechanisms controlling dynein. In drosophila wing axons LIS1 KD increased retrograde mitochondrial flux suggesting species differences that might also be found for NDEL1 (Vagnoni et al., 2016).

One caveat of our study is that we do not know the precise stoichiometry of LIS1, NDEL1, and dynein in DRG axons, and we do not know how overexpression of exogenous proteins impacts this stoichiometry. In a previous study, we estimated the molar ratio of LIS1 to dynein to be 1:28 in extracts from adult rat brain (Mesngon et al., 2006). This ratio is likely higher in axons given the importance of axonal transport and the potentially lower ratio of LIS1 to dynein in glial cells. In COS-7 cells all three overexpressed, tagged LIS1 constructs expressed at approximately the same level as endogenous LIS1 protein when using a LIS1 antibody to probe western blots (Pandey and Smith, 2011). Overexpressed WT-LIS1 appears to bind more efficiently to dynein than endogenous LIS1 so this construct may be outcompeting endogenous LIS1 for dynein binding (Pandey and Smith, 2011). The overexpressed LIS1 appears to be functional given the observed stimulated transport. R212A-LIS1 also appeared to interact more robustly with dynein. This mutant may bind to dynein without binding to NDEL1, and/or may dimerize with endogenous LIS1, which might reduce the ability of LIS1 dimers to interact with NDEL1. Either event appears to be detrimental to normal mitochondrial transport. K147A-LIS1 did not Co-IP with dynein but appeared to be binding to and sequestering endogenous LIS1 away from dynein (Pandey and Smith, 2011). Interestingly K147A-LIS1 was able to co-precipitate more myc-tagged NDEL1 than HA-tagged WT-LIS1, so this mutant may also sequester NDEL1 away from dynein. Both events could contribute to the reduced transport seen in axons when that construct is expressed. The schematics in Figures 3E–G illustrate some of the complexes that may be present in cells expressing the exogenous LIS1 constructs. Further studies will be needed to understand all of the potential dominant negative impacts the exogenous proteins have on protein interactions including those other proteins not considered here (cargo adaptors, etc.).

Another issue that will need to be addressed is the role of phosphorylation of NDEL1 by cyclin-dependent kinases on mitochondrial transport (Niethammer et al., 2000; Sasaki et al., 2000; Hebbar et al., 2008b; Pandey and Smith, 2011; Klinman et al., 2017). The role of these kinases in controlling the formation of active dynein complexes has not been explored and needs to be further investigated. There is some controversy around how CDK5 activation/inhibition impacts axonal transport. In our hands, CDK5 inhibition disrupted transport, but others have observed that CDK5 inhibition increased transport; future studies will need to address this discrepancy and clarify the role of phosphorylated and unphosphorylated NDEL1 on dynein complex formation and specific cargo transport (Pandey and Smith, 2011; Klinman and Holzbaur, 2015; Klinman et al., 2017; Chapman et al., 2019). There are also likely to be regulatory posttranslational modifications to individual polypeptides in the DDA complex itself.

The adult brain, and in particular synapses, require a large amount of energy provided mainly by mitochondria (Misgeld and Schwarz, 2017; Mandal and Drerup, 2019; Han et al., 2020). Mitochondrial recycling at synapses is thought to involve retrograde transport of spent organelles by dynein. Our inducible knockout of LIS1 in adult mice demonstrated clear postdevelopmental roles for the protein, notably regulation of axonal transport in mature sensorimotor and cardiorespiratory neurons in the hindbrain, spinal cord, and peripheral nervous system (Hines et al., 2018). Precise regulation of dynein activity by LIS1 is likely to be critical as there is evidence that increased levels of LIS1 in both humans and mice can disrupt normal brain development (Bi et al., 2009). In humans, increased LIS1 expression causes structural abnormalities in the brain, severe developmental delay, and failure to thrive (Bi et al., 2009). In mice, overexpression by about 20% caused abnormalities in the cells of the developing brain, including alterations in cellular polarity and in migration. It would be interesting to determine if induced, conditional overexpression of LIS1 in adult mice would produce neurological phenotypes like our induced KO. NDEL1 also has roles beyond embryonic development as reducing expression postnatally in forebrain excitatory neurons in mice caused spatial learning and memory deficits and seizures and short lifespan (10 weeks) in adult mice (Gavrilovici et al., 2021). Our data suggest that one possible mechanism contributing to pathologies in adult LIS1 and NDEL1 KO mice is that reduced mitochondrial recycling at synapses leads to synaptic energy deficits and compromised circuitry. Dynein has been shown to deliver the fission protein DRP 1 to mitochondria, which could be the underlying mechanism for our observation that reduced dynein function results in longer mitochondria (Varadi et al., 2004). It will be important to determine if and how LIS1 and NDEL1 impact mitochondrial fission and fusion events and the delivery of DRP1.

## Data Availability Statement

The raw data supporting the conclusions are included in the article, further inquiries can be directed to the corresponding author/s.

## Ethics Statement

The animal study was reviewed and approved by The Institutional Animal Care and Use Committee, University of South Carolina.

## Author Contributions

JP performed all experiments. LS and RB analyzed the data and edited the manuscript. DS planned the study and wrote the manuscript. All authors contributed to the article and approved the submitted version.

## Conflict of Interest

The authors declare that the research was conducted in the absence of any commercial or financial relationships that could be construed as a potential conflict of interest.

## Publisher’s Note

All claims expressed in this article are solely those of the authors and do not necessarily represent those of their affiliated organizations, or those of the publisher, the editors and the reviewers. Any product that may be evaluated in this article, or claim that may be made by its manufacturer, is not guaranteed or endorsed by the publisher.
